# Relationship between lipoprotein(a) and whole blood reducing viscosity: A cross-sectional study

**DOI:** 10.1097/MD.0000000000036236

**Published:** 2023-12-01

**Authors:** Sheng Jing, Haibo Zhu

**Affiliations:** a The First Affiliated Hospital of Ningbo University, Ningbo, China.

**Keywords:** blood viscosity, Lp(a), whole blood reduced viscosity

## Abstract

Lipoprotein(a) [Lp(a)] has been confirmed as a causal risk factor of atherosclerotic cardiovascular disease, but its role on circulation is not completely clear and is still being explored. Therefore, this study attempts to explore the relationship between Lp(a) and whole blood reducing viscosity (WBRV), to better understand the role of Lp(a) in circulatory and cardiovascular diseases. We retrospectively analyzed the data of consecutive subjects in the physical examination center of the Affiliated Hospital of Ningbo University Medical College from January 2022 to May 2022. Pearson or spearman correlation analysis was used to test the statistical relationship between 2 continuous variables according to whether they are normal; 131 participants were retrospectively enrolled in this study. The low-density lipoprotein concentration was associated with whole blood viscosity at low-shear (*R* = 0.220, *P* = .012), middle-shear (*R* = 0.226, *P* = .01), and high-shear viscosity (*R* = 0.212, *P* = .015), as well as plasma viscosity (*R*_S_ = 0.207, *P* = .018). Lp(a) was not associated with whole blood viscosity at low, middle, and high shear rates, but was associated with WBRV at low shear (*R*_S_ = 0.204, *P* = .019) and middle shear rates (*R*_S_ = 0.197, *P* = .024). Lp(a) is associated with high WBRV, which may impart more insights into the role of Lp(a) in cardiovascular disease.

## 1. Introduction

Lipoprotein(a) (Lp(a)) has been confirmed as a causal risk factor of atherosclerotic cardiovascular disease.^[1–3]^ Blood viscosity (BV) is also considered to be related to atherosclerosis and microvascular function.^[4,5]^ Contrary to expectation, many studies have shown that there is no correlation between Lp(a) and BV or plasma viscosity.^[6,7]^ However, the correlation between Lp(a) and whole blood reduced viscosity has not been concerned. In this study, we explored the relationship between Lp(a) and reduced BV.

## 2. Material and methods

### 2.1. Study population

From January 2022 to May 2022, consecutive patients who completed physical examination in the Physical examination Center of the First Affiliated Hospital of Ningbo University were retrospectively reviewed. Inclusion criteria: Patients’ physical examination items included BV and Lp(a). Patients with incomplete data were excluded. The studies involving human participants were reviewed and approved by medical ethics committee of Affiliated Hospital of Medical College of Ningbo University. Written informed consent for participation was waived for this study by medical ethics committee of Affiliated Hospital of Medical College of Ningbo University due to the retrospective nature of the study. The formulation of this study scheme was in accordance with the requirements of the Declaration of Helsinki of the World Medical Association.

### 2.2. Methods

Weight and height were measured using a standardized approach, followed by the calculation of body mass index in kg/m^2^. Venous blood was collected after overnight fasting (>8 h). BV was measured using an automated blood rheology analyzer (ZONCI Technology Co., Ltd., Beijing, China). BV levels at 5 s^−1^ are reported as low-shear viscosity, BV measurements at 30 s^−1^ are reported as middle-shear viscosity, and BV measurements at 200 s^−1^ are reported as high-shear viscosity. For plasma viscosity the average of measurements at shear rates of 200 s^−1^ was calculated.

Whole blood reductive viscosity (WBRV) refers to the whole BV value when the hematocrit (HCT) is 1, also known as the unit HCT viscosity, or is defined as the contribution of the unit HCT to the relative viscosity of the whole blood.

Whole blood reduction viscosity was calculated by the following formula:


$$\begin{array}{l} WBRV=\frac{WBV\text{\textasciitilde{}}\text{-}PV}{PV}*\frac{1}{HCT} \end{array}$$

WBV-PV is the increase of viscosity in plasma due to the addition of human blood cells, so (WBV-PV)/PV is the increase rate of viscosity increase to original viscosity. The higher of the ratio, the greater the influence of red blood cells on BV. The volume of red blood cells in the whole blood reflects the concentration of red blood cells, and its volume percentage is generally called HCT. The ratio divided by HCT is converted into the influence of unit HCT on BV. According to the formula, the whole blood reduced high shear viscosity, whole blood reduced middle shear viscosity, and whole blood reduced low shear viscosity can be calculated. Obviously, WBRV is a dimensionless value without units.

### 2.3. Statistical analysis

All statistical analyses were performed using SPSS 26.0 for Mac (IBM Corp., Armonk, NY) and GraphPad Prism version 9.0 for Mac (GraphPad Software, San Diego, CA). Data were checked for normality with the Kolmogorov–Smirnov test and shown as mean ± SD or median [P25–P75] depending on the distribution. Categorical variables are displayed as percentages and absolute numbers. Select Pearson or spearman correlation analysis to test the statistical relationship between 2 continuous variables according to whether they are normal. Level of significance was set at *P* < .05.

## 3. Results

Overall, 131 participants were enrolled in this study. Table 1 shows the characteristics of the examined population. The mean age was 64 years, mean body mass index was 25.32 kg/m^2^, and 81% were men. Lp(a) ranged from 15 to 773 mmol/L.

**Table 1:** Characteristics of the study participants.

Items	
Male, n (%)	81 (61.8)
BMI (kg/m^2^)	25.32 ± 3.46
Age (year)	64 [59–69]
NRBC (10^6^/µL)	4.79 ± 0.43
HGB (g/L)	147.76 ± 13.62
HCT (%)	43.62 ± 3.8
WBV at LSR (mPa·s)	8.69 ± 0.89
WBV at MSR (mPa·s)	5.03 ± 0.48
WBV at HSR (mPa·s)	3.78 ± 0.38
PV (mPa·s)	1.41 [1.38–1.43]
WBRV at LSR	41.74 ± 5.29
WBRV at MSR	8.32 ± 0.85
WBRV at HSR	5.45 ± 0.66
Lp(a) (mmol/L)	130 [69–273]
High-density lipoprotein (mmol/L)	1.42 ± 0.37
Low-density lipoprotein (mmol/L)	2.89 ± 0.93
Total cholesterol (mmol/L)	5.13 ± 1.21
Triglycerides (mmol/L)	1.37 [0.93–1.93]

BMI = body mass index, HCT = hematocrit, HGB = hemoglobin, HSR = high shear rate, Lp(a) = lipoprotein(a), LSR = low shear rate, MSR = middle shear rate, NRBC = RBC count, PV = plasma viscosity, WBRV = whole blood reduced viscosity, WBV = whole blood viscosity.

**Figure 1.**
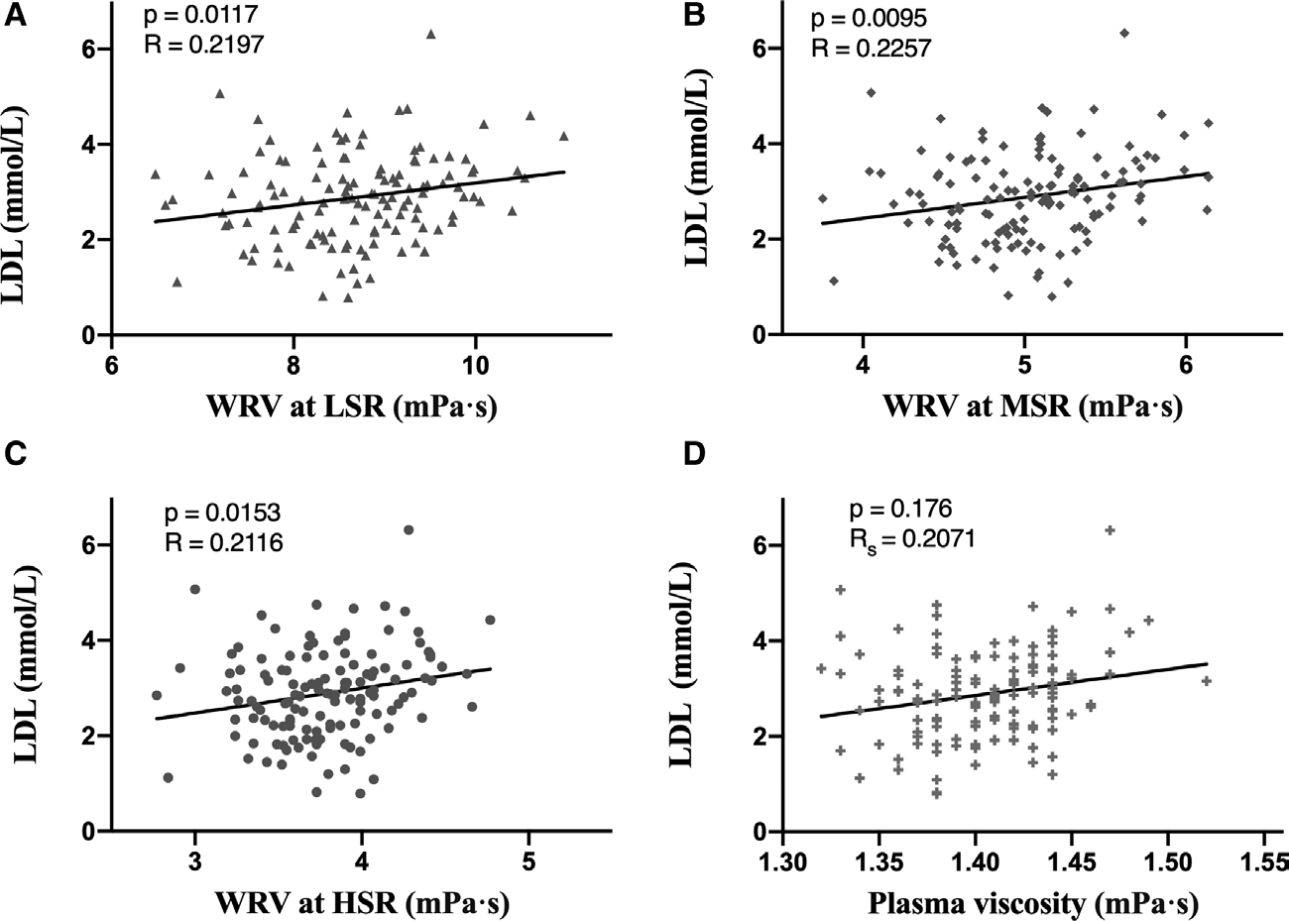
Relationship between LDL and WRV at low-shear (A), middle-shear (B), and high-shear (C), as well as plasma viscosity (D). LDL = low-density lipoprotein, WRV = whole blood reduced viscosity.

**Figure 2.**
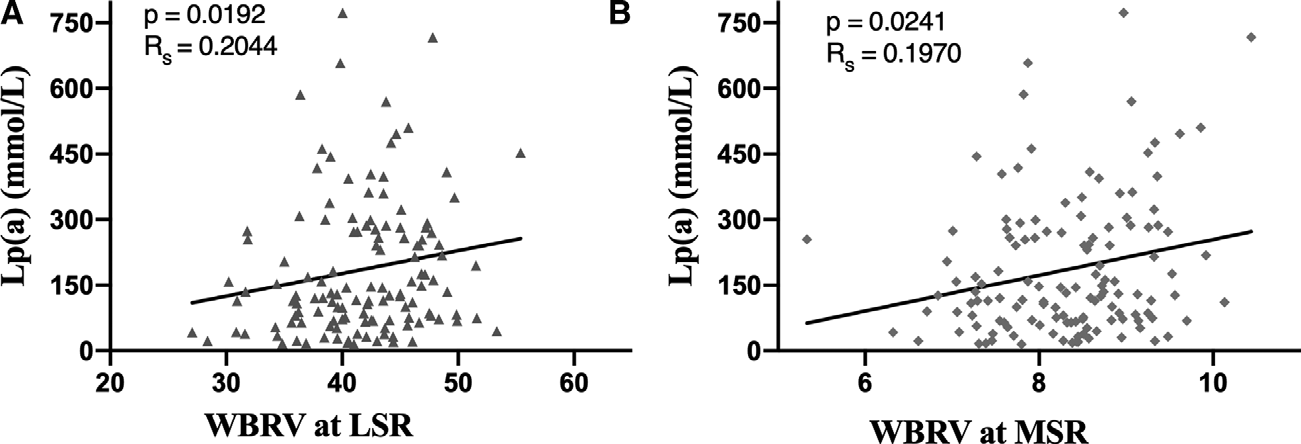
Relationship between Lp(a) and WBRV at low-shear (A) and middle-shear (B). Lp(a) = lipoprotein(a), WBRV = whole blood reducing viscosity.

High-density lipoprotein, total cholesterol, and triglycerides were not associated with whole BV, whole blood reduced BV, or plasma viscosity (Table 2). The low-density lipoprotein (LDL) concentration was associated with WBV at low-shear (*R* = 0.220, *P* = .012), middle-shear (*R* = 0.226, *P* = .01), and high-shear viscosity (*R* = 0.212, *P* = .015), as well as PV (*R*_S_ = 0.207, *P* = .018) (Fig. 1). Lp(a) was not associated with WBV at low, middle, and high shear rates, but was associated with WBRV at low shear (*R*_S_ = 0.204, *P* = .019) and middle shear rates (*R*_S_ = 0.197, *P* = .024) (Fig. 2).

**Table 2:** Association between blood viscosity and lipids (*P* values)*.

	WBV			WBRV			PV
	Low shear	Middle shear	High shear	Low shear	Middle shear	High shear	
Lp(a)	.685	.711	.839	.019	.024	.151	.05
HDL	.560	.383	.323	.899	.313	.226	.736
LDL	.012	.010	.015	.631	.448	.427	.018
Total cholesterol	.236	.180	.180	.859	.986	.880	.124
Triglycerides	.274	.338	.416	.096	.094	.247	.645

HDL = high-density lipoprotein, LDL = low-density lipoprotein, Lp(a) = lipoprotein(a), PV = plasma viscosity, WBRV = whole blood reduced viscosity, WBV = whole blood viscosity.

* This table shows the *P* values associated with viscosity and lipid variables.

## 4. Discussion

In this cross-sectional analysis, we report that LDL concentrations are associated with higher WBV measured at low-shear, middle-shear, and high-shear rates. In comparison to the lack of association between Lp(a) and WBV, concentrations of Lp(a) were associated with WBRV at low-shear and middle-shear rates.

It has long been believed that the main physiological determinants of whole blood rheology tend to be the HCT and the fibrinogen concentration. Many recent studies have shown that lipid levels are important for hemorheology.^[8]^ Specifically, the increase of high-density lipoprotein is related to the decrease of BV, while the increase of LDL and triglyceride leads to the opposite phenomenon.^[9]^ In a prior cross-sectional study, patients with type IIa hyperlipoproteinemia, a positive correlation was shown between LDL concentrations and plasma viscosity (*P* < .01).^[10]^ Another study showed that, compared to normolipidemic subjects, the PV (*P* = .007) and WBV (*P* = .013) of hyperlipidemic subjects were higher, and it was also found that PV was directly associated with LDL cholesterol in simple correlation analysis.^[7,11]^ BV depends on HCT and is a function of HCT. WBRV is defined as the influence of unit HCT on BV. In this study, we found the correlation between Lp(a) and reducing viscosity, not viscosity.

Lp(a) is a LDL cholesterol-like particle bound to apolipoprotein(a) and has been confirmed as a causal risk factor of atherosclerotic cardiovascular disease by genome-wide association, epidemiology, and clinical studies.^[12]^ In addition, there is an increasing body of evidence identifying Lp(a) as a strong, independent, and potentially causal risk factor for calcific aortic valve stenosis.^[13,14]^ To our knowledge, this is the first study that explores the relationship between WBRV and Lp(a).

Our study had several limitations. First, it is a cross-sectional study and cannot make a causal analysis. Second, data such as fibrinogen that will affect BV have not obtained due to the retrospective nature; the results may be biased. Third, due to the inclusion of a physical examination population and the retrospective nature, we were unable to obtain patient medication history and other risk factors.

## 5. Conclusion

Lp(a) is associated with high WBRV, which may impart more insights into the role of Lp(a) in cardiovascular disease.

## Acknowledgments

The authors would like to thank all the medical staff working in the cardiology department of the hospital for their support and cooperation.

## Author contributions

**Conceptualization:** Haibo Zhu.

**Data curation:** Sheng Jing.

**Formal analysis:** Sheng Jing.

**Investigation:** Sheng Jing, Haibo Zhu.

**Methodology:** Sheng Jing, Haibo Zhu.

**Project administration:** Sheng Jing, Haibo Zhu.

**Resources:** Sheng Jing.

**Software:** Sheng Jing.

**Supervision:** Haibo Zhu.

**Writing – original draft:** Sheng Jing.

**Writing – review & editing:** Haibo Zhu.
